# Spatial Analysis of Industrial Benzene Emissions and Cancer Incidence Rates in Texas

**DOI:** 10.3390/ijerph16152627

**Published:** 2019-07-24

**Authors:** Chinmay Mungi, Dejian Lai, Xianglin L. Du

**Affiliations:** 1 Department of Biostatistics and Data Science, The University of Texas Health Science Center at Houston School of Public Health, 1200 Pressler St., Room 1008, Houston, TX 77030, USA; 2 Department of Epidemiology, Human Genetics and Environmental Sciences, The University of Texas Health Science Center at Houston, 1200 Pressler St., Room 1008, Houston, TX 77030, USA

**Keywords:** benzene emission, cancer incidence, kriging, Spatial Linear Models, Spatial Pattern Analysis

## Abstract

This paper presents a spatial analysis of the association between industrial benzene emissions and the 10-year incidence rates of cancers likely to be associated with benzene exposure (Lymphohematopoietic, lung and lip cancers) at the county level in Texas. The spatial distribution of incident cases of the above cancers between 2004 and 2013 was assessed at the county level and found to have positive spatial auto-correlation. Subsequently, point pattern analysis was performed on industrial emissions of benzene reported to the Toxic Release Inventory (TRI), revealing a non-random spatial pattern. Universal kriging was performed using the industrial emissions data to derive estimates of ambient benzene levels at the county level. An ordinary linear regression model was fitted using the incidence rates as the outcome and the estimated benzene level along with chosen covariates and the residuals were assessed for lingering spatial auto-correlation. As the residuals showed that spatial auto-correlation persists, a spatial conditional auto-regression (CAR) model was fitted instead. In the spatial CAR linear regression model, estimated levels of ambient benzene were not found to be significantly associated with the 10-year incidence rates of lymphohematopoietic, lung and lip cancers at the county level.

## 1. Introduction

Cancer continues to be one of the leading causes of death both worldwide and in the United States. While incidence and mortality rates are both dropping in the United States, primarily attributed to reductions in smoking, the burden of the disease remains extremely high. The American Cancer Society estimates that 1,685,210 new cases were diagnosed in 2016 alone, and 595,690 patients succumbed to the illness [1]. 

Several natural and artificial substances have been found to be carcinogenic in humans. Benzene, a colorless liquid compound historically used in the printing industry as a component of inks as well as being a starting material in the chemical and drug industries (as a component in rubbers, lubricants, dyes, detergents, and pesticides) has been implicated in the causation of several types of leukemia and other blood-related cancers [2]. Since being identified as a human carcinogen, non-industrial use of benzene has been limited. However, industrial processes continue to release large amounts of benzene into the atmosphere, creating potential exposures for workers and the public at large. 

In recent years, researchers have begun using newer techniques to estimate the effect that exposure to ambient air pollutants, including benzene, has on the health of the general population. Mirzahosseini & Atabi [3] conducted a GIS-based study estimating the environmental benzene levels in Tehran, Iran. The methodology that the authors used was based on collecting samples at several locations around the city using a portable air monitoring device, and using those values to estimate ambient benzene levels around the city using the inverse weighted distance (IWD) technique. The authors found that the ambient levels of benzene in Tehran’s air were between 2 and 20 times higher than the international standard (1.56 ppb). They also estimated the excess cancer risk imposed by the heightened benzene levels in different neighborhoods in Tehran using an excess risk model developed by the EPA. The study falls short, however, in investigating the association between benzene levels and observed cancer incidence or mortality, due to the unavailability of cancer outcomes data containing geographical markers. 

Eiten et al [4] performed a spatial analysis of air pollution and cancer incidence rates in the Haifa Bay region in Israel. The exposures of interest in this study were PM_10_, resulting from vehicle traffic and industrial processes, as well as SO_2_, a marker of industrial emissions, while the outcomes of interest were lung cancer, bladder cancer, and non-Hodgkin’s lymphoma. Using data from Israel’s national cancer registry, the authors analyzed 143 residential wards (neighborhoods) in Haifa Bay and initially demonstrated that cancers did not follow a spatially random distribution. Subsequently, they used kriging to extrapolate region-wide estimates of the study exposures based on values from 17 SO_2_ monitoring stations and 8 PM_10_ monitoring stations within the study area. For each ward being analyzed, the average of the kriged estimates was used as the exposure level for the individuals who resided in each ward. The authors reported that SO_2_ was not associated with any of the three types of cancer, but PM_10_ showed a significant association with lung cancer in males, with each additional microgram in the environment associated with a 12% increase in lung cancer risk. 

The use of conventional linear regression models, such as Ordinary Least Squares (OLS) linear regression, presents the same problem of spatially autocorrelated residuals, violating the key assumption of independent and identically distributed error structure and alternative methods of testing the significance of any risk factors are necessary [5]. Statisticians have developed spatial regression models that are analogous to those models previously used for time-series analysis, which allowed for the correlation (in this case, between neighboring regions rather than subsequent time periods) in the outcome data [6]. This approach, which includes both simultaneous and conditional auto-regression models, allows modelling the observed values as the realizations of a distribution that depends on the values seen in neighboring areas [6]. 

Finally, the problem of using observed values to extrapolate over larger areas has been addressed using a method called kriging, introduced by Georges Matheron, who is considered the founder of geostatistical theory [5]. Kriging is a minimum-mean-squared-error used to predict spatial values based on the distribution of the observed values [6]. Various types of kriging have been developed, including simple kriging (analogous to linear prediction), ordinary kriging (based on a hypothetical mean that is common across the study region), universal kriging (where the mean structure is non-stationary, i.e. varying based on location) and block kriging (used to predict lattice data from geospatial observations). Universal kriging has been widely used in the applied literature to perform extrapolation of geographical phenomena such as soil salinity and groundwater levels [7,8], as well as man-made phenomena such as air pollution [3,4], and will be used in this study to extrapolate ambient benzene levels.

Benzene remains an important component of many industrial processes, and is released into the environment in large quantities [9]. Although EPA classifies benzene as a *harmful air particle* (HAP) and monitors levels using HAP monitor stations, none of these stations are in Texas. Individuals that reside or work in industrial areas experience prolonged exposure to high levels of ambient benzene [3]. By investigating the association between ambient benzene levels and cancer mortality, we can quantify the deleterious effects of environmental benzene exposure and help inform the public as well as policy-makers so that the necessary precautions can be taken regarding acceptable levels of ambient benzene. 

A wealth of data on socio-economic, environmental, and lifestyle determinants of health exists at the county level for Texas [10]. Furthermore, cancer incidence data for each county in the state is made publicly available [11]. Using these data, this study establishes, firstly, that incidence rates of lymphohematopoietic (leukemia, myeloma, lymphoma), lung and lip cancers in Texas between the years 2004–2013 display a pattern of spatial auto-correlation. Secondly, it shows that industrial benzene emissions in the state of Texas do not follow a spatially random pattern. Thirdly, it uses the industrial emissions data to estimate ambient benzene levels across the state of Texas. 

The primary objective was to develop a regression model that quantifies the association between benzene levels and incidence rates of cancers that are likely to be associated with benzene exposure; namely, lymphohematopoietic, lung, and lip cancers, while controlling for important covariates such as age, socio-economic status, smoking, excessive alcohol consumption, and air pollution from fine particulate matter (PM_2.5_). However, due to the spatially auto-correlated nature of the data, conventional linear regression techniques would not be appropriate, as the assumptions are not met [5]. Taking into account spatial auto-correlation, we applied a conditional autoregressive (CAR) regression model defined in next section.

## 2. Materials and Methods 

Cancer incidence data by county is provided for public use by the Texas Department of State Health Services [11]. Cases of lymphohematopoietic cancer, lung cancer, and lip cancer between 2004 and 2013 were combined to derive an overall 10-year incidence rate per county. 

**Figure 1:**
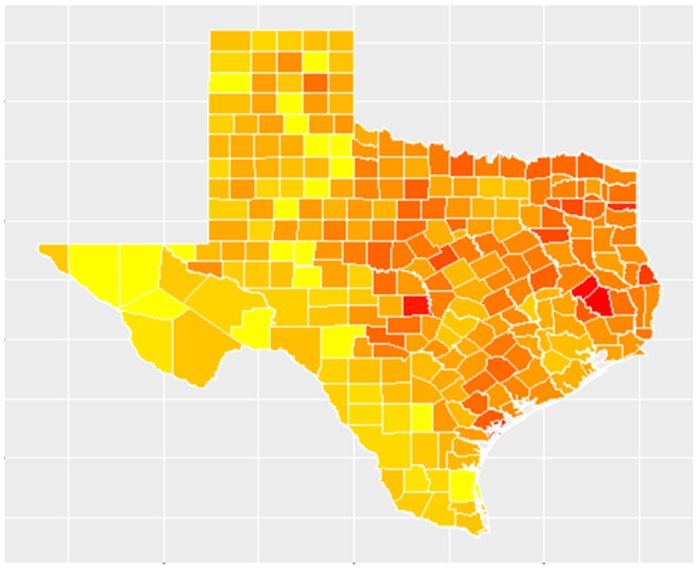
Incidence rates of benzene-associated cancers, 2004-2013.

There were 1,091,728 incident cases of invasive cancer reported to the Texas Department of State Health Services for the period spanning 2004–2013. Among these were 246,818 cases of cancers likely to be associated with benzene exposure: lymphohematopoietic, lung, and lip cancer. The highest number of these types of cancer cases was seen in Harris County with 34,999 cases of lymphohematopoietic, lung, and lip cancer over this 10-year period and 160,326 overall incident cases of cancer. The average rate of benzene-associated cancers at the county level over this 10-year period was 103.34 cases per 100,000 people. The highest rate of benzene-associated cancers was seen in Polk County with 215.78 cases per 100,000 people, whereas 12 counties did not have any incident cases of benzene-associated cancer during this period. Additionally, 8 counties in Texas do not report incident cancer cases to the Department of State Health Services. Shown in Figure 1 is a map that illustrates the spatial distribution of the 10-year incidence rates of benzene-associated cancers in Texas, ranging from lowest (in yellow) to highest (in red).

The Federal government mandates reporting of emissions data, including amounts of known dangerous chemicals released into the atmosphere for certain industries [9]. These data are released by the Environmental Protection Agency as the Toxic Release Inventory (TRI). The reported environmental releases of benzene by industrial processes during the year 2004, the beginning of the study period, were chosen to be used as a basis for estimating ambient levels of benzene. There were 149 industrial units that reported benzene emissions in 2004 in the state of Texas, of which 59 (39.6%) belong to the chemicals sector, 44 (29.5%) are from petroleum bulk terminals, and 35 (23.5%) belong to the petroleum refining sector. The remaining 11 units belonged to the hazardous waste, transportation equipment, and metal and mineral processing sectors. 

In 2004, 99, 9362.4 lbs (41.3% by volume) of these benzene emissions were classified as *fugitive air* emissions, i.e having been released into the atmosphere. Virtually all fugitive air benzene emissions can be attributed to the chemicals and petroleum refining sectors, which make up 55.2% and 41.6% of these emissions by volume, respectively.

Smoking, alcohol consumption, and fine particulate matter data are provided for public use by the County Health Roadmaps & Rankings, an initiative of the University of Wisconsin Population Health Institute [10]. The EPA’s Toxic Release Inventory [9] mandates that industries in certain sectors (including mining, manufacturing, and transport) report their emissions of certain chemicals, including benzene, and makes this data publicly available. Maps used for spatial analysis wered made from shapefiles provided by the US Census Bureau. 

All statistical analyses—consisting of calculating global indexes of spatial autocorrelation in cancer incidence rates, creating maps using shapefile data, point pattern analysis of industrial benzene emissions, estimation of ambient benzene levels using ordinary kriging, and regression analysis to estimate the effect of estimated ambient benzene levels on cancer mortality while controlling for important covariates were performed using R statistical software package with the following additional packages: “maptools”, “rgdal”, “ggplot2”, “plyr”, “splancs”, “spatstat”, “spdep”, “sp”, “gstat”, “magrittr”, “automap”, and “rgeos”. The code is available upon request. 

In order to establish whether a pattern of spatial auto-correlation exists (i.e. counties with lower rates are spatially located close to each other, and counties with higher rates are also located next to each other), global indexes of spatial autocorrelation were used [5]. Two such indexes and their corresponding test statistics are Moran’s I statistic [12] and Geary’s C [13]. Moran [12] uses the following formula:

(1) $$I=\left(\;\frac{1}{s^{2}}\;\right)\frac{\sum_{i=1}^{N}\sum_{j=1\;}^{N}\;w_{ij}\;\left(Y_{i}-\bar{Y}\right)\left(Y_{j}-\bar{Y}\right)}{\sum_{i=1}^{N}\sum_{j=1\;}^{N}w_{ij}}$$

where $s^{2}=\;\frac{1}{N}\;\sum_{i=1}^{N}$ ${\left(Y_{i}-\bar{Y}\right)}^{2}$.

The corresponding $w_{ij}$ value represents the spatial proximity of the *ith* and *jth* areas. Here, in order to analyze county level data, a simple *neighbor matrix* will be used as the W matrix, where the corresponding element is 1 if the two counties touch each other and 0 if they do not. 

Similarly, Geary’s C [13] statistic calculates a global index of spatial correlation by assigning weights by spatial proximity but scales this weighted average by a measure of overall variation around the mean regional observation $\bar{Y}$ [5]. The statistic is produced using the following formula:

(2) $$c=\frac{N-1}{2\;\sum_{i=1}^{N}\;{\left(Y_{i}-\bar{Y}\right)}^{2}}\;\;\frac{\sum_{i=1}^{N}\sum_{j=1\;}^{N}\;w_{ij}\;{\left(Y_{i}-Y_{j}\right)}^{2}}{\sum_{i=1}^{N}\sum_{j=1\;}^{N}w_{ij}}$$

By evaluating the significance of these test statistics, we tested whether the cancer incidence rates show a pattern of spatial auto-correlation. If the test statistic is significant, indicating that autocorrelation exists, we will know that it is important to account for this lack of independence in the outcome data when a regression model is fitted; if the spatial auto-correlation pattern does not exist, it would indicate that the incidence rate does not depend on any spatially distributed covariates. Both Moran’s I and Geary’s C are descriptive statistics to measure spatial autocorrelation of lattice type of data such as county cancer incidence rate. 

Locations of industrial benzene emission can be viewed as spatial point patterns. To assess whether these locations of industrial benzene emission follow a spatially random distribution, their distribution can be compared to a theoretical spatial distribution in which the event (industrial benzene emission) is equally likely to occur at any location within the area, i.e. complete spatial randomness resulting from a homogeneous Poisson process [6,14]. There are several descriptive statistics and hypothesis testing techniques such as the K, G, and F functions that can be used to compare the observed emissions locations and a theoretical spatially random distribution [5].

The K function, also known as the reduced second moment measure [15]. For any positive distance *h*, it is defined as

(3) $$K\left(h\right)=\frac{E\left[number\;of\;events\;within\;h\;of\;a\;randomly\;chosen\;event\right]}{\lambda }$$

And is estimated using the following formula

(4) $$\hat{K}\left(h\right)=\hat{\lambda }\frac{1}{N}\;\delta \;\left(d\left(i,j\right)<h\right)$$

where d(i,j) denotes the Euclidean distance between events *i* and *j*, and δ (d(i,j)<h) equals 1 if d(i,j) < h and 0 otherwise. $\hat{\lambda }$ is an estimate of the (constant) intensity given by

(5) $$\hat{\lambda }=\;\frac{Number\;of\;Events\;in\;A}{\left|A\right|}$$

where *A* is the study area and |*A*| denotes the area of *A*. The *K* function is calculated under the assumptions of constant intensity and takes the expectation across all events in the study area which assumes a process operating identically at all locations. Additionally, we used the *G* function, which is the cumulative distribution function of the distance of a randomly chosen event in the study area, and the nearest other event, as well as the F function which is the cumulative distribution function of the distance between a randomly chosen *point* and the nearest event [6]. The *G* function is estimated by the formula

(6) $$\hat{G}=\frac{\;\sum_{i=1}^{n}\;I\;(r_{i}\;\leq r,\;d_{i}>r)}{\;\sum_{i=1}^{n}\;I\;(d_{i}>r)}\;r\;>\;0$$

only in the fact that the *ith* observation does not refer to an event, but to a randomly chosen point within the study area, and $r_{i}\;$ and $d_{i}$ refer to the nearest-event distance and nearest-boundary distance from that random point, respectively [6]. 

We have estimated these three functions using the observed point pattern, and compared to their theoretical counterparts, generated under the assumption of spatial randomness. Intuitively, we would expect that if events are clustered together rather than randomly distributed, then the estimation of the *K* function using empirical data would show that the expected number of events within a certain radius of a randomly chosen one is consistently higher than it would be under the theoretical assumption of spatial randomness. Similarly, in a clustered spatial distribution, the estimated values of the *G* function will be consistently higher than under a homogeneous Poisson process (i.e. neighbors are closer than expected) and for the *F* function to have consistently lower values than expected (i.e. more empty space). These comparisons can be made visually by graphing the estimated *K*, *F,* and *G* functions against their theoretical counterparts generated by a Homogeneous Poisson process.

In addition to the methods of graphically comparing the observed point pattern to a theoretical, completely spatially random patter, we implemented two hypothesis testing techniques to test the null hypothesis that these points are the result of a spatially random process. The first is the Quadrat test, which divides the study area into a predetermined number of tiles [14]. A significant test statistic for the Quadrat test means that our null hypothesis of complete spatial randomness must be rejected. However, this could indicate either clustering or regularity since it will occur when the observed values are significantly lower or higher than expected. Additionally, the Spatial Scan test developed by Kulldorf [16] was used. This test treats the observed data as an unmarked point pattern, and uses a Poisson likelihood ratio test statistic to assess the null hypothesis of complete spatial randomness; the alternative hypothesis is that there is a Poisson process with intensity $\beta_{1}$ inside some circle of radius r, and another with intensity $\beta_{0}$ outside the circle. A significant test statistic in this case will provide evidence that there is at least one region with clustering within the study area.

After establishing that (i) cancer incidence rates show spatial auto-correlation and that (ii) benzene emissions in Texas do not follow a spatially random distribution, we can begin estimating ambient benzene levels across the state. 

Due to the lack of sensor data measuring benzene at the county level, universal kriging was performed on the basis of the measured levels of *fugitive air* benzene emissions from the Toxic Release Inventory data [9]. Universal kriging was chosen over ordinary kriging based on the assumption that air pollutant levels vary by location [5]. The county seat information was obtained from the County Health Roadmaps & Rankings data [11] and Google Maps was used to obtain the approximate longitude and latitude of each county seat. A kriging frame was created by expanding the longitude and latitude values of the county seats to cover the entire area of Texas, and the kriged estimates for each county seat location was incorporated into the lattice data set (containing the outcome data and covariates) at the county level.

Finally, we fitted a regression model to quantify the effect that the estimated ambient benzene level has on cancer incidence rate, while controlling for covariates believed to be important risk factors. Conventional linear regression uses the structure

(7) Y=Xβ+ε

where *Y* is the vector of outcomes, *X* is the vector of covariates, and *β* is a vector of coefficients. Since the outcome data in this study (incidence rate of lymphohematopoietic, lung, and lip cancer) is spatially auto-correlated, a conventional linear regression model is not appropriate. Due to the spatial auto-correlation, the residuals from an ordinary linear regression would also show spatial auto-correlation, violating the assumption of independent & identically distributed error terms [5]. Alternatively, we used a conditional autoregressive (CAR) mode. This formulation allows for the measuring of covariate effects while controlling for spatial dependence [16]. Alternatively, CAR models also explain the outcome at a particular location by the values of its neighbors, but does so by modelling the conditional probability distribution of each observation Y(s_i_) given the observed values of all other variables. The formulae that expresses the conditional mean and variance in a CAR model are

(8) $$E\left[Y\left(s_{i}\right)|Y_{-i}\right]=x{\left(s_{i}\right)}^{\prime }\beta +\sum_{j=1}^{N}c_{ij}$$

(9) $$Var\left[Y\left(s_{i}\right)|Y_{-i}\right]\;=\;\sigma^{2},\;i\;=\;1,\;\ldots ..\;,\;N$$

where $c_{ij}$ are the spatial dependence parameters. The CAR model allows us to investigate the association between estimated ambient benzene levels and cancer incidence rates in Texas while controlling for spatial dependence and important lifestyle and socio-economic determinants of health.

The final regression model was established using *R* function spautolm in package spdep. 

## 3. Results 

### 3.1. Spatial Auto-Correlation of Incidence Rates

These cancer incidence rates were assessed for spatial auto-correlation using the two standard tests of global auto-correlation, Moran’s I statistic [12] and Geary’s C statistic [13]. These tests were conducted using weights given by a simple *neighbor* matrix, where the corresponding element is 1 if the two counties are neighbors and 0 otherwise. The Moran’s I and Geary’s C was 0.475 and 0.503, respectively, with a *p*-value < 0.001 for both.

The results of both tests are highly significant, and we are compelled to reject the null hypothesis of spatial independence. The Moran’s I test statistic is positive, and the Geary’s C test statistic is less than 1, which are both consistent with a pattern of positive spatial auto-correlation. In other words, there is evidence that regions with higher incidence rates of benzene-associated cancers are clustered together, as are regions with the lower rates. It follows that any investigation into the risk factors associated with these types of cancer would benefit from taking into account the spatially auto-correlated nature of the outcome data.

### 3.2. Point Pattern Analysis of Industrial Benzene Emissions

**Figure 2:**
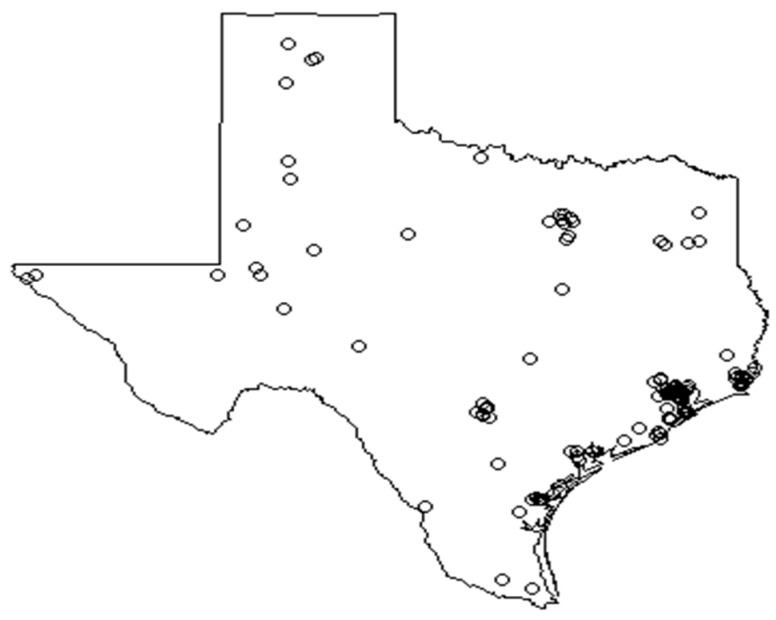
The locations of benzene emission sites.

The locations of industrial benzene emissions were assessed using point pattern analysis, in order to test whether these emissions follow a pattern of complete spatial randomness, as opposed to a pattern of clustering or regularity. Shown in Figure 2 are the locations of the benzene-emitting industrial sites in 2004, overlaid on a map of Texas.

The locations of industrial benzene emissions visually appear to be clustered in certain regions of the state, and this intuition can be confirmed statistically using the point pattern summary—*K*, *F* and *G*—functions [5] that compare the observed point pattern to a hypothetical spatially random pattern, as well as the hypothesis testing methods of the Quadrat Test [14] and the Spatial Scan Test [16].

**Figure 3:**
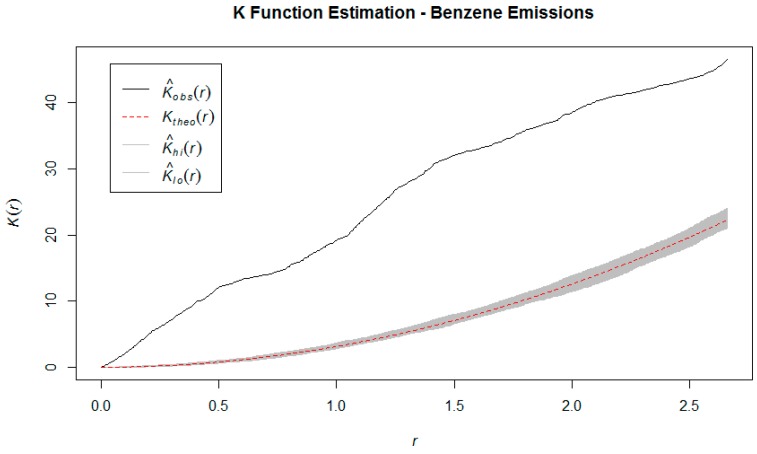
K function estimation benzene emission sites.

**Figure 4:**
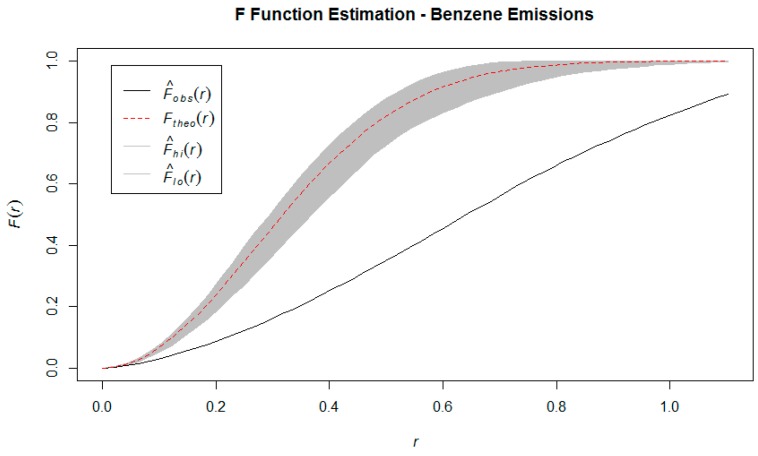
F function estimation benzene emission sites.

**Figure 5:**
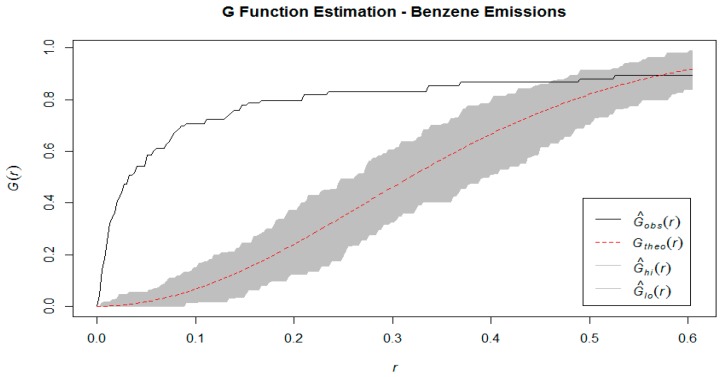
G Function estimation benzene emission sites.

From Figure 3, Figure 4 and Figure 5, we notice that the estimates of the *K*, *F,* and *G* functions are all outside the 95% confidence band. The direction of the estimates (above or below the confidence bands) indicate that the observed point pattern has more points clustered within any given radius, that there is less empty space around each point, and that neighboring points are closer to each other, when compared to a homogeneous Poisson point process (i.e. the distribution that would result in a spatially random point pattern). These findings supported the conclusion that the point pattern made up by the industrial benzene emissions follows a *cluster* pattern, as opposed to complete spatial randomness or regular spacing [6]. The Quadrat test statistic and Kulldorf’s Spatial Scan Test statistic were 523.29 (*p* < 0.001) and 358.79 (*p* < 0.001), which also indicate spatial dependence. We are compelled by the results of both these hypothesis tests to reject the null hypothesis of complete spatial randomness. Thus, there is evidence that the industrial benzene emissions data does not follow a random point pattern, but rather that the locations of these emissions are clustered together in certain regions [16]. 

### 3.3. Regression Analysis Using Kriged Estimates

Universal kriging was performed, using location and volume of the industrial benzene emissions, along with the latitude and longitude data of each county seat in Texas to obtain an estimate of the ambient benzene level in each county [5]. Initially, an ordinary linear regression model was fitted using the benzene-associated cancer incidence rates as the outcome, and the kriged estimate of ambient benzene along with the proportion of smoking, excessive drinking, individuals over 65 years of age, fine particulate matter (PM2.5) levels, and median household income (in thousands of $ per annum) as covariates. From the residuals, Moran’s I and Geary’s C were 0.174 and 0.836, respectively, with *p*-values < 0.001. These results showed that the ordinary linear regression may not be an appropriate method for modelling this association and a spatial regression model should be used instead.

Using the same covariates mentioned above, a conditional autoregressive (CAR) model was fitted with benzene-associated cancer incidence rates as the outcome. The spatial weights for this regression were once again provided using a simple *neighbor* matrix. A summary of the results is shown in Table 1. The approximate 95% confidence intervals were calculated directly using the estimates plus 1.96 times the standard error as the upper bound and the estimates minus the 1.96 times the standard error as the lower bound. 

Although simple CAR linear regression (one covariate) did show significance, the kriged estimates of ambient benzene level did not show a significant association with the 10-year incidence rates of hematological, lung and lip cancers when regressed along with the other covariates. The percentage of individuals who drink alcohol excessively, smoke, or who are over 65 years of age were found to have significant associations with these incidence rates at the county level, showing a positive relationship in each case. Additionally, the average daily levels of fine particulate matter (PM2.5) were found to have a statistically significant positive relationship with the incidence rates of these types of cancer, whereas median household income was seen to confer a significant protective effect. Subsequently, the residuals from the CAR model were tested for remaining spatial auto-correlations. The Moran’s I and Geary’s C was −0.165 and 1.176 with *p*-values = 1.0, which indicates that the spatial auto-correlation in the outcome data is explained in a satisfactory manner by the variables in the conditional auto-regression model. In a comparison, the standard error of residuals from simple regression was 35.93 and 32.64 from the CAR. This indicated that the CAR model not only improved the model fitting over the simple linear regression instead of spatial independence, but also provided smaller variability of residuals.

## 4. Discussions

This analysis demonstrates that the incidence rates of lymphohematopoietic, lung and lip cancers in Texas between 2004 and 2013 follow a non-random spatial distribution. It is therefore likely that environmental or geographical factors play a role in the etiology of these diseases. The role of environmental or geographical factors is supported by the inability of conventional linear regression methods to explain the spatial auto-correlation seen in the incidence rates in this sample. The association of air pollution and increased lung cancer risk is a prominent trend in the applied statistics literature [17]. Many researchers assert that the health effects of air pollution have been consistently underestimated in analyses that do not use a spatial framework, with the authors of a spatial analysis of air pollution and overall mortality in Los Angeles making the claim that the overall health effects of heavy air pollution may be three times as great as previously believed [18]. The patterns of spatial non-randomness seen in these outcome data are similar to those found in studies conducted in other regions, particularly with lung cancer [4]. For outcome data that shows significant spatial auto-correlation, conventional regression methods are not appropriate and regression models that allow for this auto-correlation, such as the conditional auto-regression (CAR) model used here, should be used instead [19]. 

The fact that the point pattern analysis shows that industrial emissions of benzene are clustered around the major cities and industrial sites in Texas comes as no surprise. However, the fact that these locations are also the greatest population centers in the state means that the potential deleterious effects of these industrial emissions affect a great number of people. While this model does not show a significant association between the estimated benzene levels and the incidence rates of the cancers in question, it is possible that with more accurate exposure data (e.g. from sensor stations) a different picture may emerge. A recent study on predictors of residential mobility and its impact on air pollution exposure among children diagnosed with early childhood leukemia was conducted at the household level [20].

The epidemiological literature on the carcinogenicity of benzene has historically been focused on estimating the effects of high levels of exposure, commonly seen among workers in the rubber and chemicals industries and found elevated incidence rates and mortality from blood-related cancers [21,22,23]. Subsequent researchers have revisited the original *Pliofilm* cohort, as well as conducting studies using cohorts taken from other industries, with mixed results; typically, significant associations were only seen between benzene exposure and cancer incidence or mortality in the group of workers in the highest exposure category [24,25]. Other studies found significant associations between acute benzene exposure and lung and lip cancer when using a study design that classified exposure in a categorical manner (comparing outcomes in workers that were exposed to benzene, to the outcome of workers from industries where benzene was not used) [26]. 

The results of this modelling exercise are consistent with most of the epidemiological literature, finding that low levels of ambient benzene exposure are not significantly associated with incidence rates of lymphohematopoietic, lung and lip cancers at the county level. The significant association between fine particulate matter (PM 2.5) and incidence rates of lymphohematopoietic, lung and lip cancers seen in this spatial regression is also consistent with the findings of a spatial analysis study conducted in Haifa Bay, Israel which found a significant association with this type of air pollution and incidence rates of lung and bladder cancer at the ward level [4]. 

This study suffers from three primary drawbacks that may introduce bias and affect the accuracy of the results: (i) the use of industrial emissions data as the basis for estimation (due to the unavailability of sensor data for this type of pollutant), (ii) combining three distinct types of cancer for analysis, and (iii) using the county as the unit of analysis as opposed to a smaller area more likely to have a uniform level of air pollution. 

The universal kriging performed in this model used the fugitive air emissions from industrial units, measured at the source. The estimates thus obtained are far less accurate as a measure of population exposure than estimates based on sensor data collected at ground level. Studies have been conducted using a portable photo ionization detector for the purpose of measuring benzene and other air pollutants and subsequently using these measurements to estimate pollutant levels over a larger area [3]. 

Due to the verifiable low incidence of some of the cancers at county level, we combined incidences of three cancer types (lymphohematopoietic, lung and lip cancers) and, which have been found to be associated with benzene exposure in the epidemiological literature but may not have similar etiologies, which is another potential source of bias. However, with a different study design (e.g. a spatial case/control study), a larger sample, or a longer follow-up period, more effective results could be obtained by considering each type of cancer as a distinct outcome and performing the analyses separately. 

Finally, the regression model was fitted using the county level as the unit of analysis. This was done to utilize the wealth of health and socio-economic data available at the county level in Texas. However, due to the large size of each county, the assumption that air pollution levels are the same across the county is not realistic. Spatial analysis conducted using smaller divisions of area would yield more accurate results.

## 5. Conclusions

The estimated levels of ambient benzene at the county level were not significantly associated with incidence rates of lymphohematopoietic, lung, and lip cancers in this sample. However, the accuracy of the estimates is likely to have been affected by the lack of ground-level measurements. Further study using the appropriate device to measure ground-level benzene levels and using those to estimate ambient benzene levels at the county level is warranted. 

The significant association seen between levels of fine particulate matter (PM2.5) and the incidence rates of the above cancers in this and other studies [4] is a cause for concern as this type of air pollution is associated with industrial activity and therefore present in highly populated areas, putting many people at risk. Although primarily associated with lung cancer in the literature, there have been studies that show associations between fine particulate matter air pollution and respiratory illness [27]. The associations between industrial air pollution and respiratory illness, as well as possible associations with other types of cancer, should also be investigated further using the appropriate spatial analysis framework. 

As many studies have shown before, cancer and cancer rates are associated with many confounders which could have resulted in significant spatial auto-correlation. A comprehensive review on spatial modeling in environmental and public health research was given in [28]. The authors have pointed out that it is problematic to simultaneously control for all known risk factors and researchers may have to rely on spatial methods [28]. A simple parametric model used in our study is an easy way of addressing the complicated effect of genetic and environmental human carcinogens. Taking into account the spatial auto-correlation, we did not find significant statistical association between industrial benzene emission and the county level cancer incidence rate of lymphohematopoietic (leukemia, myeloma, lymphoma), lung and lip cancers in Texas. Because this study was unable to measure benzene level at an individual level, the findings could be affected by ecological fallacy. The findings from this study do not necessarily alter the fact that benzene is a human carcinogen and industrial benzene emission should be monitored constantly at various levels to improve environmental status and health for all.

## Figures and Tables

**Table 1:** Results from Conditional Autoregressive Linear Model.

Variable	Estimated Coefficient	Std. Error	95% Confidence Interval	z-Value	Pr (>|z|)
Intercept	−236.799	62.26	(−358.829, −114.769)	−3.80	<0.001
Estimated Benzene	6.389	4.70	(−2.823, 15.601)	1.36	0.175
Percentage of excessive drinking	4.029	1.92	(0.266, 7.792)	2.10	0.035
Percentage of smokers	4.201	1.69	(0.889, 7.513)	2.48	0.013
Average daily PM2.5 level	17.601	3.29	(1.153, 24.049)	5.34	<0.001
Median Household Income	−0.857	0.30	(−1.445, −0.269)	−2.85	0.004
Percentage over 65 years of age	4.032	0.66	(2.738, 5.326)	6.12	<0.001
	Spatial dependence parameter: *ρ* = 0.131				
